# Toward the Impact of Non-pharmaceutical Interventions and Vaccination on the COVID-19 Pandemic With Time-Dependent SEIR Model

**DOI:** 10.3389/frai.2021.648579

**Published:** 2021-03-18

**Authors:** Yuexin Li, Linqiang Ge, Yang Zhou, Xuan Cao, Jingyi Zheng

**Affiliations:** ^1^Department of Mathematics and Statistics, Auburn University, Auburn, AL, United States; ^2^TSYS School of Computer Science, Columbus State University, Columbus, GA, United States; ^3^Department of Computer Science and Software Engineering, Auburn University, Auburn, AL, United States; ^4^Department of Mathematical Sciences, University of Cincinnati, Cincinnati, OH, United States

**Keywords:** COVID-19, epidemiology, dynamic modeling, reinfection, vaccination, time-dependent SEIR model

## Abstract

The outbreak of COVID-19, caused by the SARS-CoV-2 coronavirus, has been declared a pandemic by the World Health Organization (WHO) in March, 2020 and rapidly spread to over 210 countries and territories around the world. By December 24, there are over 77*M* cumulative confirmed cases with more than 1.72*M* deaths worldwide. To mathematically describe the dynamic of the COVID-19 pandemic, we propose a time-dependent SEIR model considering the incubation period. Furthermore, we take immunity, reinfection, and vaccination into account and propose the SEVIS model. Unlike the classic SIR based models with constant parameters, our dynamic models not only predicts the number of cases, but also monitors the trajectories of changing parameters, such as transmission rate, recovery rate, and the basic reproduction number. Tracking these parameters, we observe the significant decrease in the transmission rate in the U.S. after the authority announced a series of orders aiming to prevent the spread of the virus, such as closing non-essential businesses and lockdown restrictions. Months later, as restrictions being gradually lifted, we notice a new surge of infection emerges as the transmission rates show increasing trends in some states. Using our epidemiology models, people can track, timely monitor, and predict the COVID-19 pandemic with precision. To illustrate and validate our model, we use the national level data (the U.S.) and the state level data (New York and North Dakota), and the resulting relative prediction errors for the infected group and recovered group are mostly lower than 0.5%. We also simulate the long-term development of the pandemic based on our proposed models to explore when the crisis will end under certain conditions.

## 1. Introduction

**Figure 1:**
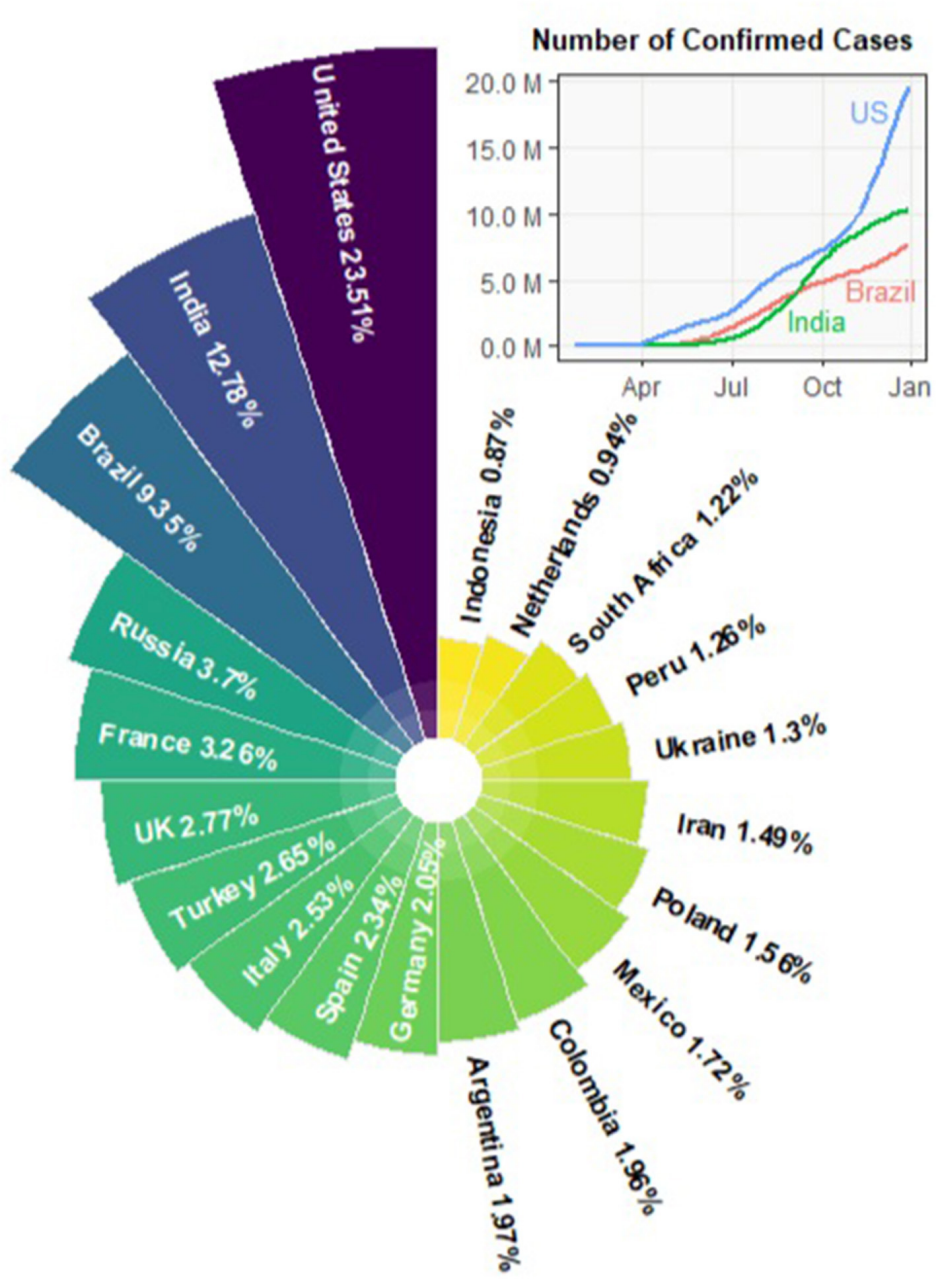
Countries most impacted by COVID-19, updated by 2020-12-24.

On March 11, 2020, the World Health Organization (WHO) declared that the outbreak of the novel coronavirus (COVID-19) can be characterized as a pandemic. The COVID-19 outbreak started in Wuhan, China in December, 2019. By the end of January, 2020, the confirmed cases in China went up to 11, 791. Only 1 month later, the number increased nearly seven-fold to 80, 134 and the COVID-19 cases gradually showed up in other countries. Starting from March, 2020, the outbreak spread to more than 100 countries. By the end of 2020, the pandemic has led to 77.5*M* confirmed cases and more than 1.72*M* fatalities worldwide. Figure 1 summarizes the percentage of global confirmed cases contributed by each country. As of December 24, the United States, India, and Brazil are the three countries most impacted by the COVID-19 pandemic. The trajectories of the confirmed cases in the three countries are also displayed.

The COVID-19 virus has caused a great disruption to the human health, social life, developments, and economics. To stop the spread of COVID-19 virus, governments have carried out numerous preventive measures such as stay-at-home orders, travel restrictions, school closure, mask-wearing mandate, and so forth. The impact on the society came later in all aspects, including rising unemployment, protests against restrictions, and psychological anxiety and stress brought to the public. However, a significant decrease in the transmission rate occurred, which proved that these mitigation measures were effective. Months later, many states in the U.S. have loosened their restrictions and lifted orders to allow businesses to reopen to the public. Consequently, the diagnoses of daily confirmed cases have displayed a consequential increasing trend after the reopen in some states such as Alabama. By looking at the numbers only, it is difficult to assess what stage we are at in the COVID-19 pandemic and when it is going to end. Hence, mathematical models considering the epidemiological characteristics of COVID-19 become crucial and significant to track and forecast the trend of the spread.

The classic epidemiology model exhibits compelling results, especially during the early period of the pandemic. The compartmental models, which are the simplified versions of mathematical models for infectious diseases, divide the population into different compartments between which people may progress. Different diseases are represented by different compartmental models (Schmidt, 1981; Sharomi and Gumel, 2011; Gao et al., 2016). The Susceptible-Infectious-Recovered (SIR) model, as one of the simplest and most classic compartmental models, characterizes the dynamic changes in each compartment using ordinary differential equations. There are three compartments in this model: susceptible (*S*), infectious (*I*), and recovered/deceased (*R*). The number of individuals in each compartment varies over time. The deterministic SIR and its derivatives are widely used to predict infectious deceases like COVID-19 (Chen et al., 2020; Katul et al., 2020; Toda, 2020). Besides compartmental models, statistical learning techniques are also widely used in biomedical fields (Zheng et al., 2018, 2019; Hsieh and Zheng, 2019; Ganyani et al., 2020; Murray, 2020; You et al., 2020). For example, IHME team (Murray, 2020) employed a statistical model to predict the number of deaths, the demand of hospital beds, ICU beds and ventilators in a few months.

In this paper, we develop a time-dependent Susceptible-Exposed-Infectious-Recovered (SEIR) model with coefficients estimated by Least Absolute Shrinkage and Selection Operator (LASSO) regression. This model is inspired by the SIR model and takes the existence of incubation period (the time from exposure to development of symptoms) into consideration. The individuals who have been infected but are not yet infectious are labeled as exposed (*E*). Instead of the constant parameters used in traditional SIR based models, we propose to model the dynamic with time-dependent parameters. Additionally, we extend our SEIR model to accommodate other crucial factors such as immunity, reinfection, and vaccination cases into account. With the epidemiology models, we aim at answering the following questions:

- What is the trajectory of transmission rate, incubation rate, and recovery rate?
- Has the inflection point been reached. If so, when?
- How does the reopen order affect the spread of the pandemic?
- How do reinfection and vaccination affect the pandemic?
- When will the mortality reach the peak?
- How many cases do we expect to have when the pandemic is over?

The remainder of the paper is organized as follows: we build the time-dependent SEIR model in section 2. Then we extend the model to include the vaccinated group as well as analyze the asymptotic stability of its disease-free equilibrium in section 3. To validate our model, we perform numerical analysis, prediction, and model simulation using national level data of the United States, and the state level data of two selected states, New York and North Dakota. The results are presented in section 4. Lastly, we conclude this paper in section 5.

## 2. The Time-Dependent SEIR Model

Our proposed SEIR model with time-dependent parameters describes the transmission dynamic of an epidemic. It is assumed that there are totally four states in which an individual would experience: susceptible, exposed, infected, and recovered. In the susceptible state, the individual does not have the disease but can be infected by someone infectious through an effective contact. Once being infected, the individual moves to the exposed state. The exposed individual is not able to infect others until the incubation period is over. Eventually, the infected individual recovers from the disease. Altogether the four groups of individuals at different states compose the entire population and we denote the number of individuals in each group at time *t* by *S*(*t*), *E*(*t*), *I*(*t*), and *R*(*t*). In this model, a person is assumed to be immune to the virus after recovery and will not return to the susceptible state. Accordingly, the number of deaths caused by the disease is also counted in the recovered group *R*(*t*) since neither of the recovered and dead has any more impact on the spread of the virus.

The differential equations that govern the trajectories of the four compartments are formulated as:

(1) $$\begin{array}{l} \frac{dS}{dt}=-\frac{\beta_{t}S\left(t\right)I\left(t\right)}{N}, \end{array}$$

(2) $$\begin{array}{l} \frac{dE}{dt}=\frac{\beta_{t}S\left(t\right)I\left(t\right)}{N}-\sigma_{t}E\left(t\right), \end{array}$$

(3) $$\begin{array}{l} \frac{dI}{dt}=\sigma_{t}E\left(t\right)-\gamma_{t}I\left(t\right), \end{array}$$

(4) $$\begin{array}{l} \frac{dR}{dt}=\gamma_{t}I\left(t\right), \end{array}$$

with a constant total population *N*,

(5) $$\begin{array}{l} N=S\left(t\right)+E\left(t\right)+I\left(t\right)+R\left(t\right), \end{array}$$

and therefore, we have:

(6) $$\begin{array}{l} \frac{dS}{dt}+\frac{dE}{dt}+\frac{dI}{dt}+\frac{dR}{dt}=0. \end{array}$$

Three time-dependent parameters, the transmission rate β_*t*_, the transition rate σ_*t*_, and the recovery rate γ_*t*_ are introduced in this model, which are all assumed to vary with respect to time. The descriptions and empirical ranges are listed in Table 1.

**Table 1:** Model parameters.

**Parameter**	**Description**	**Empirical range**	**References**
			
β_*t*_	Transmission rate (effective contact rate) at a given time	0.5–1.5 day^−1^	Ngonghala et al., 2020; Read et al., 2020; Shen et al., 2020
σ_*t*_	Transition rate from exposed to infections at a given time	$\frac{1}{5.1}$	Fairoza Amira et al., 2020; Ngonghala et al., 2020
γ_*t*_	Recovery rate at a given time	$\frac{1}{10}$	Fairoza Amira et al., 2020; Ngonghala et al., 2020
*v*_*t*_	Fraction of susceptible individuals vaccinated at a given time		
*w*	Fraction of infections gain immunity after recovery		

The proportion of susceptible and infected individuals in the population at time *t* are $\frac{S\left(t\right)}{N}$ and $\frac{I\left(t\right)}{N}$, respectively. Given the transmission rate β_*t*_, which describes the flow of susceptible becoming exposed to the virus, and the total population *N*, the number of newly exposed people is $\frac{\beta_{t}S\left(t\right)I\left(t\right)}{N}$. Later, the exposed individuals make the transition to the infected state at the transition rate σ_*t*_, which is the inverse of the incubation period. The number of exposed individuals who complete the transition at time *t* is σ_*t*_*E*(*t*). Similarly, people recovered at time *t* is γ_*t*_*I*(*t*), given the recovery rate γ_*t*_, which is the number of individuals recover from the infected state per person per time.

### 2.1. Discrete Time-Dependent SEIR Model

Since the COVID-19 case report is updated daily, we revise the differential Equations (1)–(4) into discrete time difference equations as follows:

(7) $$\begin{array}{l} S\left(t+1\right)-S\left(t\right)=-\frac{\beta_{t}S\left(t\right)I\left(t\right)}{N}, \end{array}$$

(8) $$\begin{array}{l} E\left(t+1\right)-E\left(t\right)=\frac{\beta_{t}S\left(t\right)I\left(t\right)}{N}-\sigma_{t}E\left(t\right), \end{array}$$

(9) $$\begin{array}{l} I\left(t+1\right)-I\left(t\right)=\sigma_{t}E\left(t\right)-\gamma_{t}I\left(t\right), \end{array}$$

(10) $$\begin{array}{l} R\left(t+1\right)-R\left(t\right)=\gamma_{t}I\left(t\right), \end{array}$$

with the four variables satisfying (5) and

(11) $$\begin{array}{l} S\left(t+1\right)-S\left(t\right)+E\left(t+1\right)-E\left(t\right)+\\ I\left(t+1\right)-I\left(t\right)+R\left(t+1\right)-R\left(t\right)=0. \end{array}$$

Assuming historical data for a certain time period 0 ≤ *t* ≤ *T* is available, i.e., we have {*S*(*t*), *E*(*t*), *I*(*t*), *R*(*t*)|0 ≤ *t* ≤ *T*}. By deduction from (7) to (10), we can compute historical values of the parameter series {β_*t*_, σ_*t*_, γ_*t*_|0 ≤ *t* ≤ *T* − 1} using the following formulas:

(12) $$\begin{array}{l} \beta_{t}=\frac{N\left(E\left(t+1\right)-E\left(t\right)+I\left(t+1\right)-I\left(t\right)+R\left(t+1\right)-R\left(t\right)\right)}{S\left(t\right)I\left(t\right)}, \end{array}$$

(13) $$\begin{array}{l} \sigma_{t}=\frac{I\left(t+1\right)-I\left(t\right)+R\left(t+1\right)-R\left(t\right)}{E\left(t\right)}, \end{array}$$

(14) $$\begin{array}{l} \gamma_{t}=\frac{R\left(t+1\right)-R\left(t\right)}{I\left(t\right)}. \end{array}$$

Now predicting future values of the parameters {β_*t*_, σ_*t*_, γ_*t*_|*t* ≥ *T*} given historical values can be converted to a regression problem.

### 2.2. Tracking the Transmission Rate β_*t*_, Transition Rate σ_*t*_, and Recovery Rate γ_*t*_

There are several approaches predicting future values of the time-dependent parameters. For instance, we can use linear models (e.g., linear regression), nonlinear methods (e.g., spline), or time series models (e.g., autoregressive model), etc. In this subsection, we fit the following LASSO regression models:

(15) $$\begin{array}{l} {\hat{\beta }}_{t+1}=a_{0}+\overset{I}{\underset{i=1}{\sum }}a_{i}\beta_{t-i}, \end{array}$$

(16) $$\begin{array}{l} {\hat{\sigma }}_{t+1}=b_{0}+\overset{J}{\underset{j=1}{\sum }}a_{j}\sigma_{t-j}, \end{array}$$

(17) $$\begin{array}{l} {\hat{\gamma }}_{t+1}=c_{0}+\overset{K}{\underset{k=1}{\sum }}a_{k}\gamma_{t-k}, \end{array}$$

where *I*, *J*, and *K* are the orders of the autoregressive process, and {*a*_*i*_|0 ≤ *i* ≤ *I*}, {*b*_*j*_|0 ≤ *j* ≤ *J*} and {*c*_*k*_|0 ≤ *k* ≤ *K*} are the regression coefficients.

These coefficients are determined by minimizing the following loss functions, which are composed of the residual sums of squares (RSS) and regularization terms:

(18) $$\begin{array}{l} L\left(\beta \right)=\overset{T-1}{\underset{t=I+1}{\sum }}{\left(\beta_{t}-a_{0}-\overset{I}{\underset{i=1}{\sum }}a_{i}\beta_{t-i}\right)}^{2}+\lambda_{\beta }\overset{I}{\underset{i=0}{\sum }}|a_{i}^{2}|, \end{array}$$

(19) $$\begin{array}{l} L\left(\sigma \right)=\overset{T-1}{\underset{t=J+1}{\sum }}{\left(\sigma_{t}-b_{0}-\overset{J}{\underset{j=1}{\sum }}b_{j}\sigma_{t-j}\right)}^{2}+\lambda_{\sigma }\overset{J}{\underset{j=0}{\sum }}|b_{j}^{2}|, \end{array}$$

(20) $$\begin{array}{l} L\left(\gamma \right)=\overset{T-1}{\underset{t=K+1}{\sum }}{\left(\gamma_{t}-c_{0}-\overset{K}{\underset{k=1}{\sum }}c_{k}\gamma_{t-k}\right)}^{2}+\lambda_{\gamma }\overset{K}{\underset{k=0}{\sum }}|c_{k}^{2}|, \end{array}$$

λ_β_, λ_σ_, and λ_γ_ are the regularization parameters deciding the penalty to the flexibility of model, and all regularization parameters can be optimized by cross-validation.

### 2.3. Estimating the Exposed $\hat{E}$(*t*), Infections $\hat{I}$(*t*), and Recovered $\hat{R}\left(t\right)$ Groups

Given the historical data {*S*(*t*), *E*(*t*), *I*(*t*), *R*(*t*), 0 ≤ *t* ≤ *T*}, we first compute the time-dependent parameter series {β_*t*_, σ_*t*_, γ_*t*_, 0 ≤ *t* ≤ *T* − 1} introduced in section 2.1. Then we predict future values $\left\{{\hat{\beta }}_{t},{\hat{\sigma }}_{t},{\hat{\gamma }}_{t},t\geq T\right\}$ using the model built in section 2.2. According to (8), (9), (10), and (5), we can further predict the number of cases for the future as follows:

(21) $$\begin{array}{l} \hat{E}\left(t+1\right)=\hat{E}\left(t\right)+\frac{{\hat{\beta }}_{t}\hat{S}\left(t\right)\hat{I}\left(t\right)}{N}-{\hat{\sigma }}_{t}\hat{E}\left(t\right),\;t\geq T+1, \end{array}$$

(22) $$\begin{array}{l} \hat{I}\left(t+1\right)=\hat{I}\left(t\right)+{\hat{\sigma }}_{t}\hat{E}\left(t\right)-{\hat{\gamma }}_{t}\hat{I}\left(t\right),\;t\geq T+1, \end{array}$$

(23) $$\begin{array}{l} \hat{R}\left(t+1\right)=\hat{R}\left(t\right)+{\hat{\gamma }}_{t}\hat{I}\left(t\right),\;t\geq T+1, \end{array}$$

(24) $$\begin{array}{l} \hat{S}\left(t+1\right)=N-\hat{E}\left(t+1\right)-\hat{I}\left(t+1\right)-\hat{R}\left(t+1\right),\;t\geq T+1, \end{array}$$

Note that for the special case when estimating $\left\{\hat{S}\left(t\right),\hat{E}\left(t\right),\hat{I}\left(t\right),\hat{R}\left(t\right)|t=T+1\right\}$, i.e., the numbers of cases at *t* = *T*+1, we use the true values of {*S*(*t*), *E*(*t*), *I*(*t*), *R*(*t*)|*t* = *T*} instead of using the estimated values $\left\{\hat{S}\left(t\right),\hat{E}\left(t\right),\hat{I}\left(t\right),\hat{R}\left(t\right)|t=T\right\}$ as in the formulas (21), (22), (23), and (24). The detailed steps of the entire procedure are summarized in Algorithm 1.

**Algorithm 1:**
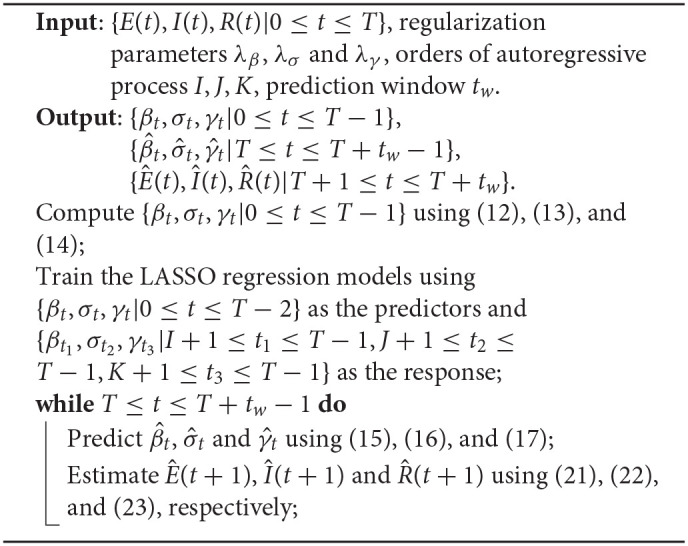
Tracking discrete time time-dependent SEIR model

## 3. SEIR Variation Considering Immunity, Reinfection, and Vaccination

The human immune system protects the body against diseases with two parts. The first part, known as the innate immune response, includes the release of chemicals that cause inflammation and white blood cells that can destroy infected cells. It is always ready to take actions as soon as any foreign invader is detected inside the body. However, this part is not specific to coronavirus. It will not learn and develop immunity to the virus. Instead, the second part: the adaptive immune response produces targeted antibodies that can stick to the virus and stop the spread to the body. The T cells^1^ would attack the cells infected by the virus.

Existing research shows that most COVID-19 patients had an antibody response at 10 days or later after onset of symptoms (To et al., 2020). If the adaptive immune response is powerful enough, it could leave a lasting memory of the infection that will provide protection in the future. Other findings also suggest that strong responders (with higher antibody level) are significantly higher in severe patients, while it is unclear whether the asymptomatic or mildly symptomatic patients will develop sufficient adaptive immune response and gain immunity to the disease after recovery (Tan et al., 2020). In fact, there have been several reported cases of COVID reinfection in China, Hong kong, Belgium, the Netherlands, and the U.S. (Tan et al., 2020), and the reinfection case are indeed increasing. This implies the necessity of taking reinfection into consideration.

On the other hand, the worldwide endeavor to create a safe and effective COVID-19 vaccine is beginning to bear fruit. A wide variety of vaccines has already been authorized around the globe while many more remain in development. According to the U.S. CDC, as of December 13, 2020, the Pfizer-BioNTech COVID-19 vaccine has been authorized and large-scale (Phase 3) clinical trials are in progress or being planned for three other vaccines in the United States. Currently the supply of COVID-19 vaccine in the U.S. is limited, but it will increase in the upcoming weeks and months. Once large quantities are available, the increasingly large-scale vaccination will have a substantial impact on the pandemic.

### 3.1. The Time-Dependent SEVIS Model

To take the factors of immunity, reinfection, and vaccination into account, we modify the proposed SEIR model by removing the recovered group *R*(*t*) and adding a vaccinated group *V*(*t*), which represents the vaccinated individuals. In this susceptible, exposed, vaccinated, and infected modeling framework, the previous assumption for the SEIR model that an infected individual will not become susceptible again after recovery is no longer employed. Instead, we assume that a fraction of the infected individuals gain immunity after recovery through producing antibodies while the rest return to the susceptible state. The former is counted in the *V*(*t*) group along with the vaccinated individuals since, epidemiologically speaking, both are immune to the virus and can no longer be infected. The new SEVIS model is governed by the following differential equations:

(25) $$\begin{array}{l} \;\frac{dS}{dt}=-\frac{\beta_{t}S\left(t\right)I\left(t\right)}{N}-v_{t}S+\left(1-w\right)\gamma_{t}I\left(t\right), \end{array}$$

(26) $$\begin{array}{l} \frac{dE}{dt}=\frac{\beta_{t}S\left(t\right)I\left(t\right)}{N}-\sigma_{t}E\left(t\right), \end{array}$$

(27) $$\begin{array}{l} \frac{dV}{dt}=v_{t}S+w\gamma_{t}I\left(t\right), \end{array}$$

(28) $$\begin{array}{l} \frac{dI}{dt}=\sigma_{t}E\left(t\right)-\gamma_{t}I\left(t\right), \end{array}$$

with a constant total population *N*,

(29) $$\begin{array}{l} N=S\left(t\right)+E\left(t\right)+V\left(t\right)+I\left(t\right), \end{array}$$

and therefore, we have:

(30) $$\begin{array}{l} \frac{dS}{dt}+\frac{dE}{dt}+\frac{dV}{dt}+\frac{dI}{dt}=0. \end{array}$$

The parameter settings of the transmission rate β_*t*_, the transition rate σ_*t*_, and the recovery rate γ_*t*_ remain the same as in the SEIR model. The vaccination rate *v*_*t*_ is low at the beginning of vaccine administration and gradually increasing as supply is growing. *w* ∈ [0, 1] is the fraction of infected cases that become immune after recovery. In addition, we assume it to be constant in this model. Hence, the number of infected individuals recover at time *t* is γ_*t*_*I*(*t*), and *wγ*_*t*_*I*(*t*) join the *V*(*t*) group while (1 − *w*)γ_*t*_*I*(*t*) fail to gain immunity and return to the susceptible state *S*(*t*).

### 3.2. Baseline Epidemiological Parameters

In previous studies, the transmission rate, β (as a constant), ranges from around 0.5 to 1.5 per person per day (Ngonghala et al., 2020; Read et al., 2020; Shen et al., 2020) and decreases as time goes. Based on existing literature, the incubation period (the time from exposure to development of symptoms) of COVID-19 and other coronaviruses ranges from 2 to 14 days. On average, symptoms show up in the newly exposed person about 5.1 days after contact (Fairoza Amira et al., 2020; Ngonghala et al., 2020). Thus, the transition rate, which is the inverse of the incubation period, is estimated to be $\frac{1}{5.1}$.

### 3.3. Basic Reproduction Number and Asymptotic Stability of Disease-Free Equilibrium

In this subsection we give the closed-form expression for the time-dependent basic reproduction number of the SEVIS model using the next generation operator method (Diekmann et al., 1990; van den Driessche and Watmough, 2002). The basic reproduction number $R_{0}$ is defined as the average number of secondary infections caused by a single infectious individual who enters an entirely susceptible population. That actually is the special case where all parameters and compartments are at their initial state at time *t* = 0. Since we propose the parameters to be time-dependent in our model, we revise the basic reproduction number to a time-dependent version $R_{t}$ as well. When $R_{t}>1$, the infection will be able to start spreading in the population and develop into an epidemic. Generally speaking, it is more difficult to control the epidemic with the larger the value of the basic reproduction number.

Let *X* be the vector of infected classes and *Y* be the vector of uninfected classes. For the SEVIS model (25)–(28), we have:

$$\begin{array}{l} X=\left[\begin{array}{c} E\\ I \end{array}\right],Y=\left[\begin{array}{c} S\\ V \end{array}\right]. \end{array}$$

Next we define the matrix of new infection terms F, which only includes the flow from *X* to *Y*, and matrix of all other terms V, which includes flows within *X* and flows leaving the system. For each compartment, in-flow in V is negative and out-flow in V is positive.

$$\begin{array}{l} F=\left[\begin{array}{c} \frac{\beta_{t}SI}{N}\\ 0 \end{array}\right],V=\left[\begin{array}{c} \sigma_{t}E\\ -\sigma_{t}E+\gamma_{t}I \end{array}\right]. \end{array}$$

The next generation matrix is defined as *FV*^−1^ where:

$${F=\frac{\partial F}{\partial X}|}_{DFE},{V=\frac{\partial V}{\partial X}|}_{DFE}.$$

The disease-free equilibrium (DFE) of the SEVIS model is given by: (S^*^, E^*^, V^*^, I^*^) = (*N*, 0, 0, 0), and we have

$$\begin{array}{l} F=\left[\begin{array}{cc} 0 & \beta_{t}\\ 0 & 0 \end{array}\right],V=\left[\begin{array}{cc} \sigma_{t} & 0\\ -\sigma_{t} & \gamma_{t} \end{array}\right]. \end{array}$$

Therefore, the next generation matrix is:

$$\begin{array}{l} FV^{-1}=\left[\begin{array}{cc} \beta_{t}/\gamma_{t} & \beta_{t}/\gamma_{t}\\ 0 & 0 \end{array}\right]. \end{array}$$

$R_{t}$, the basic reproduction number at time *t*, is given by the dominant eigenvalue of *FV*^−1^:

(31) $$\begin{array}{l} R_{t}=\frac{1}{2}\left(\frac{\beta_{t}}{\gamma_{t}}+\sqrt{\frac{\beta_{t}}{\gamma_{t}}\left(\frac{\beta_{t}}{\gamma_{t}}+4\right)}\right). \end{array}$$

Similarly, we can obtain the same basic reproduction number for the time-dependent SEIR model. The DFE is locally asymptotically stable if $R_{t}<1$, and unstable if $R_{t}>1$.

## 4. Numerical Results, Predictions, and Simulations

In this section, we will give the numeric results obtained by implementing Algorithm 1 on the national level data of the United States (US) as well as the state level data of a few representative states.

**Figure 2:**
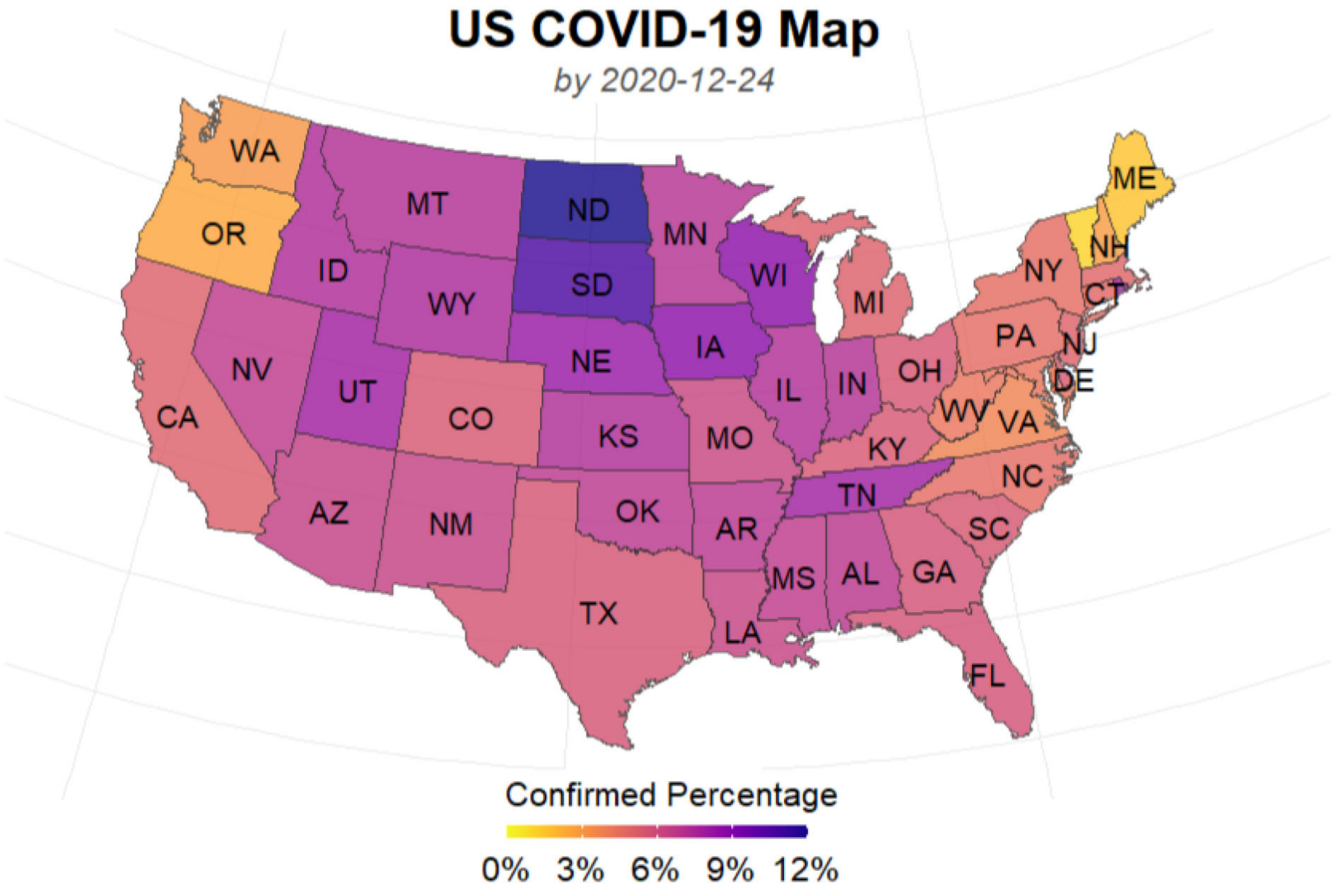
U.S. COVID-19 map.

In spring 2020, the New York Metropolitan Area experienced the largest COVID-19 outbreaks. As thousands of cases were being confirmed daily in New York, the state was the epicenter of the nation's crisis and on a different scale than the rest of the country. Though some new batches of hotspots have emerged across the country during the past months, the state of New York (NY) is still a region worth studying. On the other hand, as of December 24, a pack of northern states close to the Canada-US border have the highest percentages of cumulative confirmed cases in their populations as shown in Figure 2. The top one, North Dakota, has 11.94% of its population infected cumulatively, followed by South Dakota (10.69%), Wisconsin (8.61%), and some other nearby states. In this case, as a representative of this particular area, we take North Dakota (ND) as another example to illustrate our algorithm. We used the dataset that was collected from the COVID-19 data repository by the Center for Systems Science and Engineering (CSSE) at Johns Hopkins University (Dong et al., 2020) and the **nCov2019** R package (Wu et al., 2020). The dataset contains time series of the numbers of confirmed cases, recovered cases and deaths up to December 24, 2020. The starting date of the training set used for model training varies according to the actual spread of the pandemic in each of the three regions: US, NY, and ND. For each region, a different start date of training set is chosen for model fitting according to the time when a relatively clear trend emerges.

**Figure 3:**
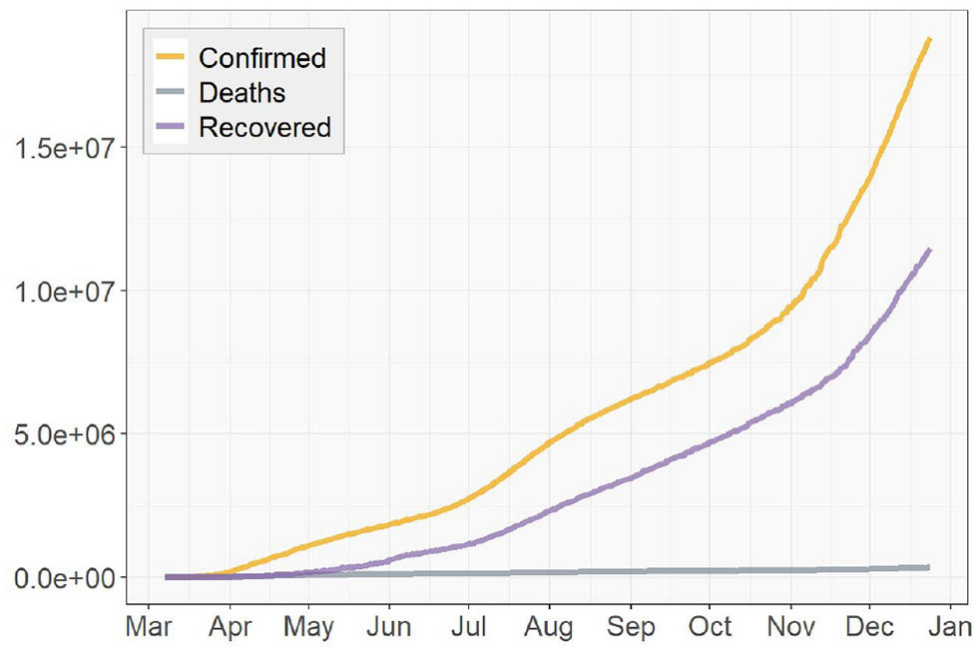
Cumulative numbers of COVID-19 confirmed cases, recoveries, and deaths in the United States.

**Figure 4:**
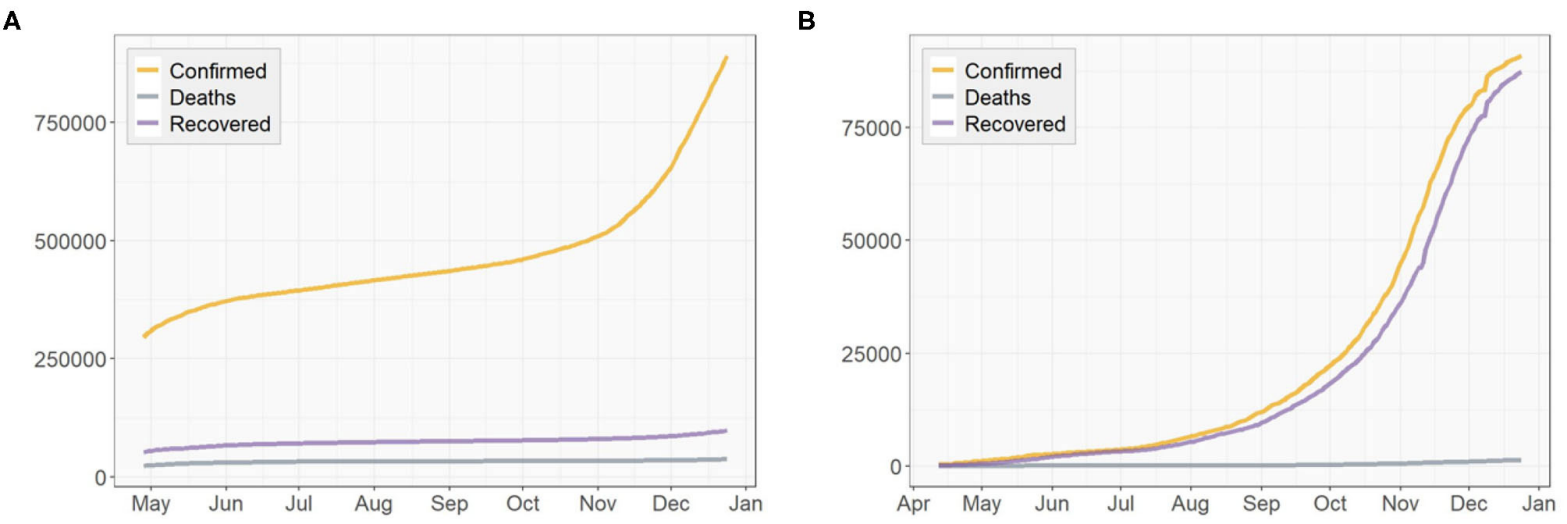
Cumulative numbers of COVID-19 confirmed cases, recoveries, and deaths in **(A)** New York, **(B)** North Dakota.

Figures 3, 4 presents the cumulative numbers of COVID-19 confirmed cases, recoveries and deaths reported in US, NY, and ND. The data starts at the beginning of the pandemic for US and ND, but it starts a while after the initial point for NY. The reason is that, back when the pandemic first started, a series of well-recorded numbers of recoveries were not available for many states, including NY. To obtain complete data on the three type of cases for computation, a cut-off is made. Therefore, the starting point of the data we collected for NY is about 2 months later than the actual date when the first case of COVID-19 was confirmed.

Due to the unavailability of the numbers of the exposed individuals *E*(*t*) in any of these regions, we substitute our model in section 2.1 with a simplified version as in Chen et al. (2020) that only includes the other three compartments *S*(*t*), *I*(*t*), and *R*(*t*). To validate our algorithm, we compare the prediction results with known data to see how well it performs, or how large the prediction errors are. Then we implement the algorithm again to predict how the COVID-19 pandemic will spread in the future.

At the end of this section, we simulate the long-term development of the pandemic based on the epidemiology models proposed in sections 2, 3 by constructing certain conditions and assigning assumed values to the parameters listed in Table 1. Based on the results, we discuss what they indicate as well as what differences we expect to see in reality compared to the simulation.

### 4.1. Parameter Tracking and Prediction

First we compute the true values of the transmission rate β_*t*_ and the recovery rate γ_*t*_ using (12), (13), and (14). Then starting from the sixth day in the parameter series, we take the value of a time-dependent parameter for each day as a subject for testing and a 5-day window before it as a corresponding observation used for training, i.e., *I, K* = 5 in section 2.2. By doing this, we construct the training and testing sets for model fitting. The R package **glmnet** is used to fit the LASSO regression models and choose the optimal values of λ_β_ and λ_γ_ that yield the minimum mean cross-validated errors.

**Figure 5:**
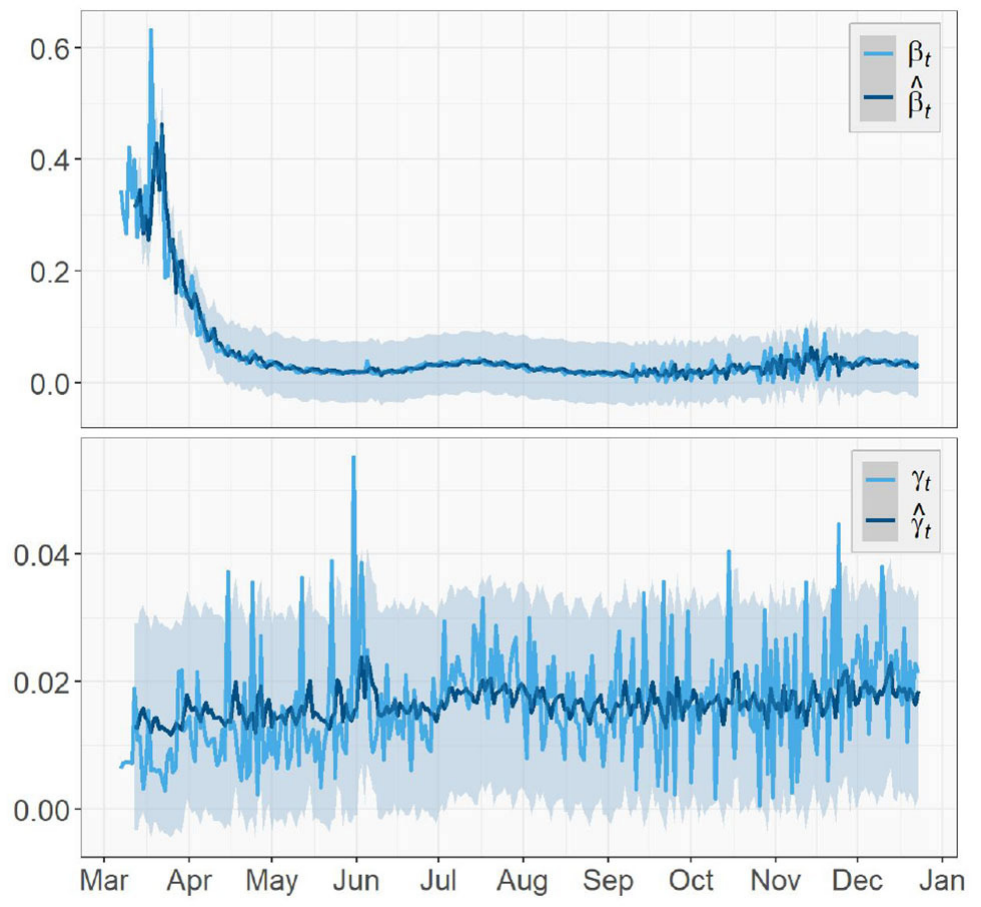
Parameter tracking and prediction for the United States.

**Figure 6:**
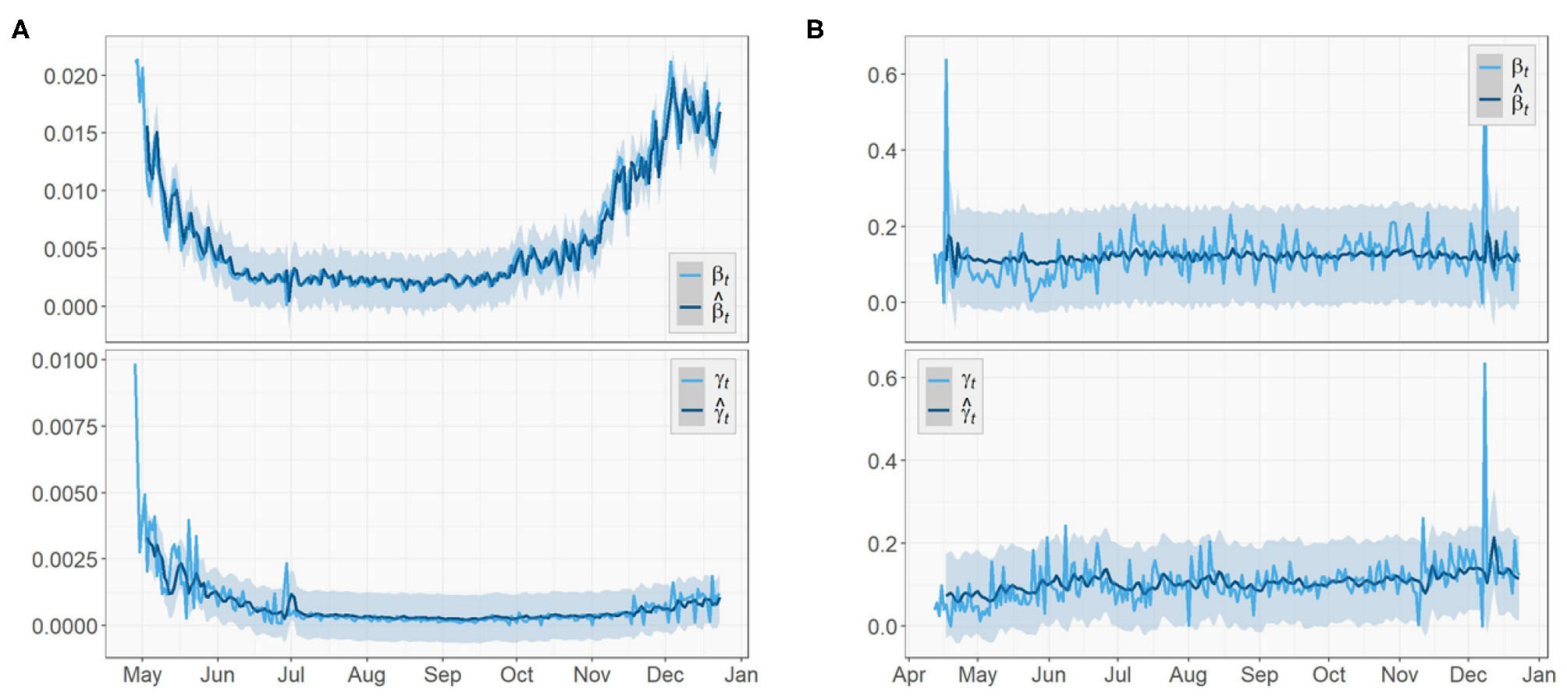
Parameters tracking and prediction for **(A)** New York **(B)** North Dakota.

Figures 5, 6 depict the true values {β_*t*_, γ_*t*_|0 ≤ *t* ≤ *T* − 1} and predicted values $\left\{{\hat{\beta }}_{t_{1}},{\hat{\gamma }}_{t_{2}}|I+1\leq t_{1}\leq T-1,K+1\leq t_{2}\leq T-1\right\}$ of both the transmission rate and the recovery rate of US, NY, and ND, respectively. The 95% prediction intervals are shown as the gray bands above and below the curves.

For the U.S. case, there was a sharp decrease in the transmission rate from mid-March to May, just about 1 week after the spread of the virus started. This was an evidence that the social distancing measures and community lockdowns implemented across the country have effectively and significantly slowed down the spread of the pandemic. It kept decreasing for about a month before a surge appeared in July, which is possibly caused by the nationwide celebration of Independence Day. In the fall, starting from early September, the transmission rate slowly rose again with increasingly larger oscillations, which showed consistency with the surge in the fall that pushed the total number of confirmed cases in US past 11*M*. This could be a result of a series of events prior to that (e.g., school opening, Halloween), and a prelude to the upcoming large gathering (e.g., Thanksgiving, Black Friday, Christmas). We expect this increase in the transmission rate to continue toward early 2021 and start to gradually decrease after the vaccination is administrated at a large scale in U.S. The recovery rate also had an slight increase around the same time in July but not as large as the one in the transmission rate. Overall, the recovery rate of U.S. is relatively steady and does not show any significant increasing or decreasing trend.

Similar to the US case, the transmission rate of NY started high and then reduced rapidly in the next few weeks. The trend maintained stationary for about 3 months until a rise appeared in late September and kept increasing toward the end. By December, the transmission rate is nearly as high as when it first started. The recovery rate of NY also had a large initial value followed by a 2-month-long decrease, but no clear trend was shown after a small spike at the beginning of July.

As for the ND case, the recovery rate started with a mild increase in the first 2 month. Later on, it remained steady just like the previous two regions. For the transmission rate, the overall trend is much more stationary compared to the results of US and NY and no significant change could be observed. However, the true values of the two parameters of ND have the greatest oscillations, i.e., the largest ranges of oscillations, among the three regions. Note the two unusually acute spikes in the transmission rate respectively in May and December and one in the recovery rate in December that deviate from the entire curves. In the absence of any pre or post trend, we consider these points as outliers in this paper and exclude them in model training.

### 4.2. Algorithm Validation and Relative Percentage Errors

In this section, we use the computed values of the parameters to estimate the three variables *S*(*t*), *I*(*t*), and *R*(*t*) as in section 2.3. Instead of directly predicting future values for *t* > *T*, we use the historical data {*I*(*t*), *R*(*t*)|*T* − *t*_*w*_ ≤ *t* ≤ *T* − 1} and the predicted parameter series $\left\{{\hat{\beta }}_{t},{\hat{\gamma }}_{t}|T-t_{w}\leq t\leq T-1\right\}$ to estimate the last *t*_*w*_ days of the entire period of time by which the data is covered, i.e., predict $\left\{\hat{I}\left(t\right),\hat{R}\left(t\right)|T-t_{w}+1\leq t\leq T\right\}$. Moreover, we also compare the proposed model with the classic SIR model with constant parameters by replacing the time-dependent parameter series with their means.

We evaluate the model performance using the relative percentage errors (*RPE*) of the prediction for the infected group *I*(*t*) and the recovered group *R*(*t*) as follows:

(32) $$\begin{array}{l} RPE_{I}=\frac{\left|I\left(t\right)-\hat{I}\left(t\right)\right|}{I\left(t\right)},\;T-t_{w}+1\leq t\leq T, \end{array}$$

(33) $$\begin{array}{l} RPE_{R}=\frac{\left|R\left(t\right)-\hat{R}\left(t\right)\right|}{R\left(t\right)},\;T-t_{w}+1\leq t\leq T. \end{array}$$

**Figure 7:**
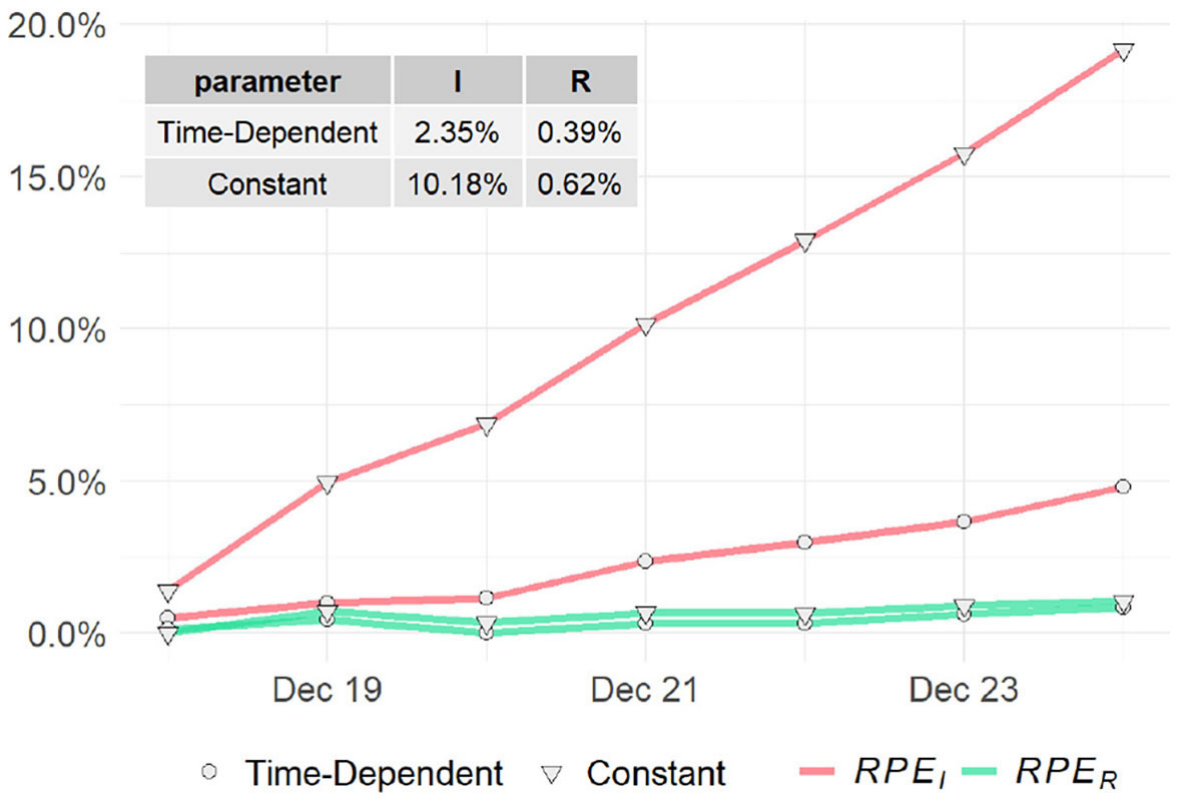
Relative prediction errors for the United States.

**Figure 8:**
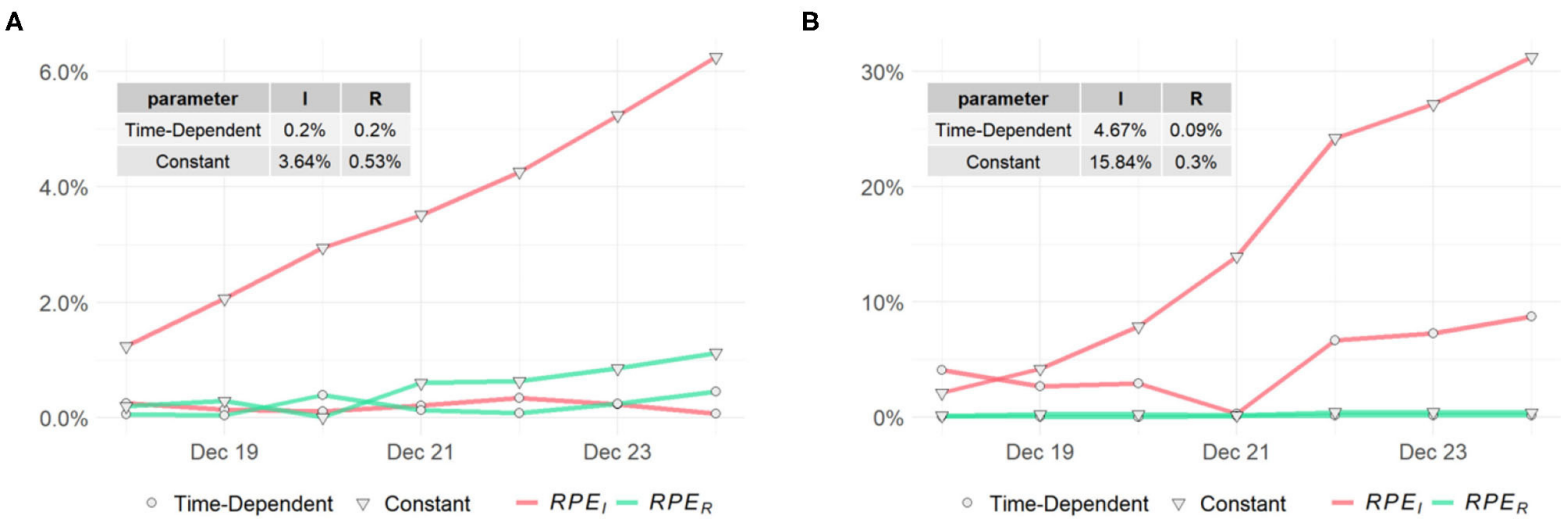
Relative prediction errors for **(A)** New York **(B)** North Dakota.

To assess the predictions of the proposed method and compare with the classic SIR model, we compute the *RPE* series for the past week (i.e., *t*_*w*_ = 7) for the two models. The *RPE* series for US, NY, and ND are displayed in Figures 7, 8 respectively, with their means summarized in the top-left corner of each figure. Using the proposed model with time-dependent parameters, the mean relative percentage errors for *I*(*t*) and *R*(*t*), i.e., *RPE*_*I*_ and *RPE*_*R*_, are 2.35 and 0.39% for US, 0.2 and 0.2% for NY, and 4.67 and 0.09% for ND, respectively. Using the classic SIR model with constant parameters, *RPE*_*I*_ and *RPE*_*R*_ are 10.18 and 0.62% for US, 3.64 and 0.53% for NY, and 15.84 and 0.3% for ND, respectively. All errors are significantly larger than the former, which clearly shows the proposed time-dependent model yields better results in predicting the spread of the pandemic than the traditional SIR model with fixed parameters. Details of the model training and validation process are summarized in Table 2.

**Table 2:** Modeling training and validation.

**Region**	**Start date of training data**	**Size of training set**	**Order**	**Prediction window *t*_*w*_**	**Mean *RPE*_*I*_ (%)**	**Mean *RPE*_*R*_ (%)**
United States	2020−03−07	287	5	7	2.35	0.39
New York	2020−04−28	235	5	7	0.2	0.2
North Dakota	2020−04−12	251	5	7	4.67	0.09

### 4.3. One-Day Prediction for *I*(*t*), *R*(*t*), and Basic Reproduction Numbers

**Figure 9:**
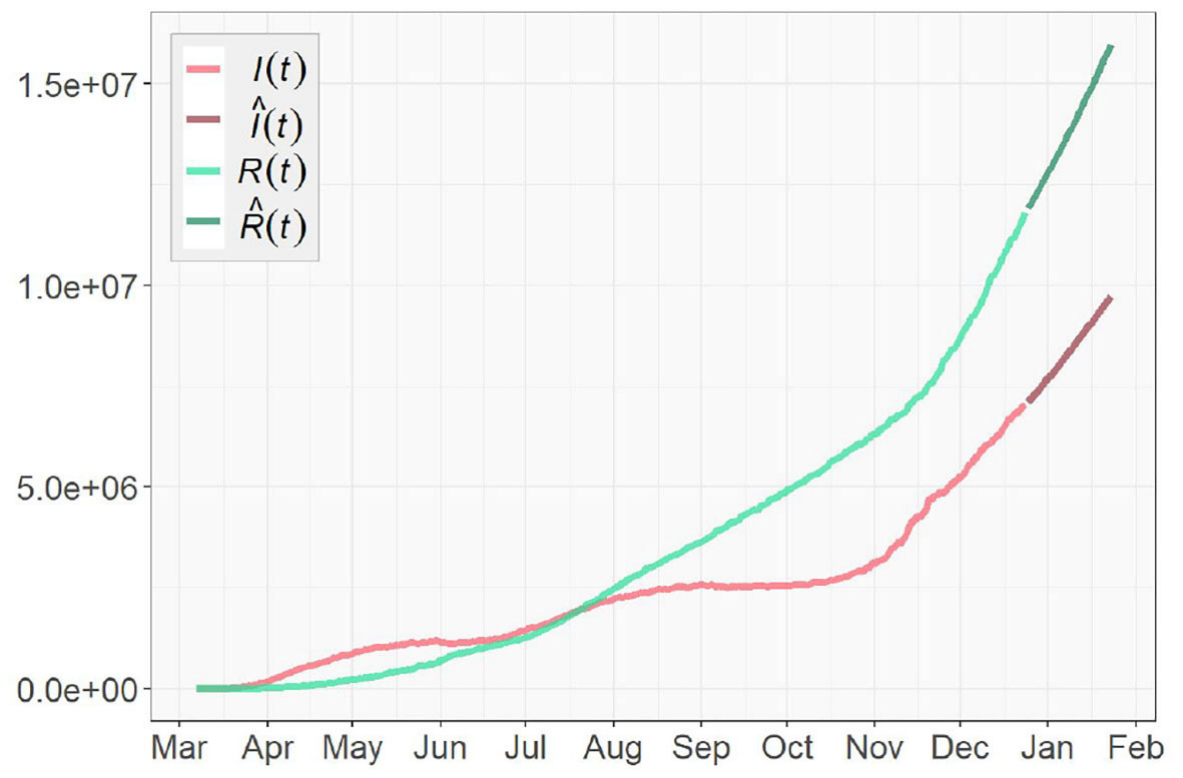
One-day prediction of 30 days for the United States.

**Figure 10:**
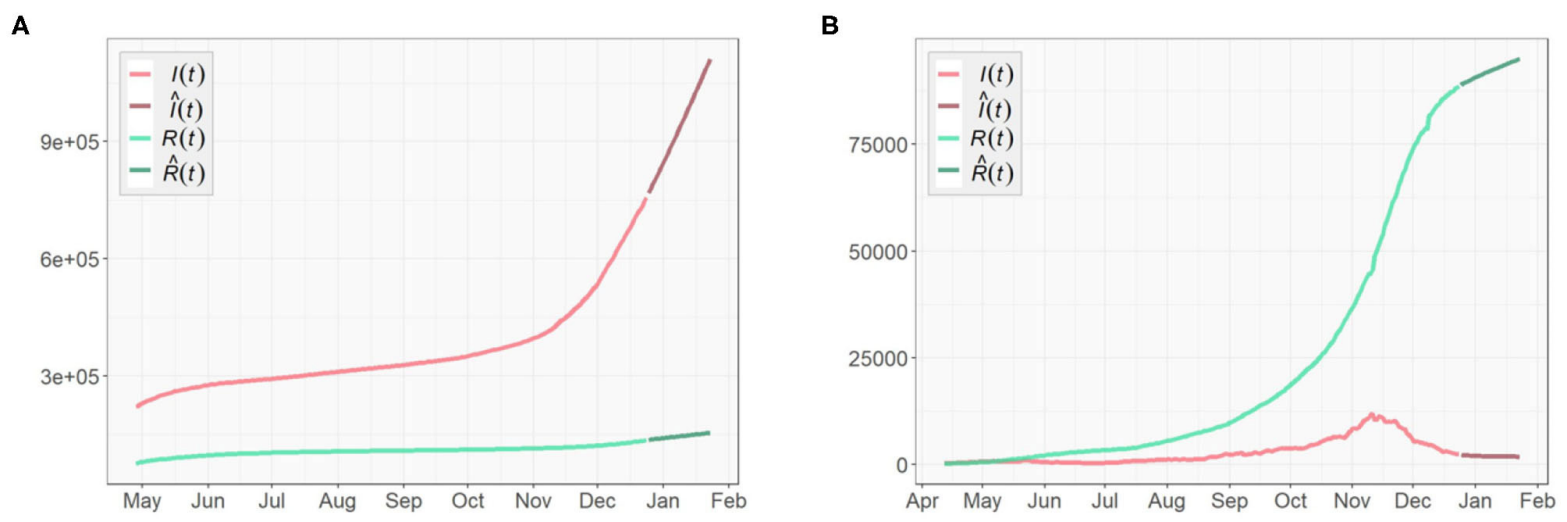
One-day prediction of 30 days for **(A)** New York **(B)** North Dakota.

Next we implement Algorithm 1 to predict the number of infected *I*(*t*) and recovered individuals *R*(*t*) for the future $\left\{\hat{I}\left(t\right),\hat{R}\left(t\right)|T+1\leq t\leq T+t_{w}\right\}$. We reset the prediction window *t*_*w*_ to be 30, as we are to predict the spread of COVID-19 pandemic in the next 30 days after December 24, 2020. The results of 1-day prediction for US, NY, and ND are shown in Figures 9, 10, respectively. For NY, the sharp increase in the infected group since November is predicted to continue toward the next year, due to the oscillatory rise in the transmission rate shown in Figure 6. On the other hand, the growth of the recovered group remains slow. For ND, the number of infected will stay low after the small surge was contained in November, while the rapid growth in the recovered group is expected to be continuous but might slow down. For US, the prediction shows that both curves will keep climbing at a high rate, which indicates that there will still be a long way to go before the pandemic finally ends. The prediction results are summarized in Table 3.

**Table 3:** Prediction results.

**Region**	**Total confirmed cases on last day**	**Prediction window *t*_*w*_**	****$\hat{I}$(*t*)****	**$\hat{R}\left(t\right)$**	**Predicted total confirmed cases**
United States	18,829,816	30	9,723,682	15,971,038	25,694,720
New York	891,270	30	1,111,117	153,277	1,264,394
North Dakota	90,947	30	1,760	95,016	96,776

**Figure 11:**
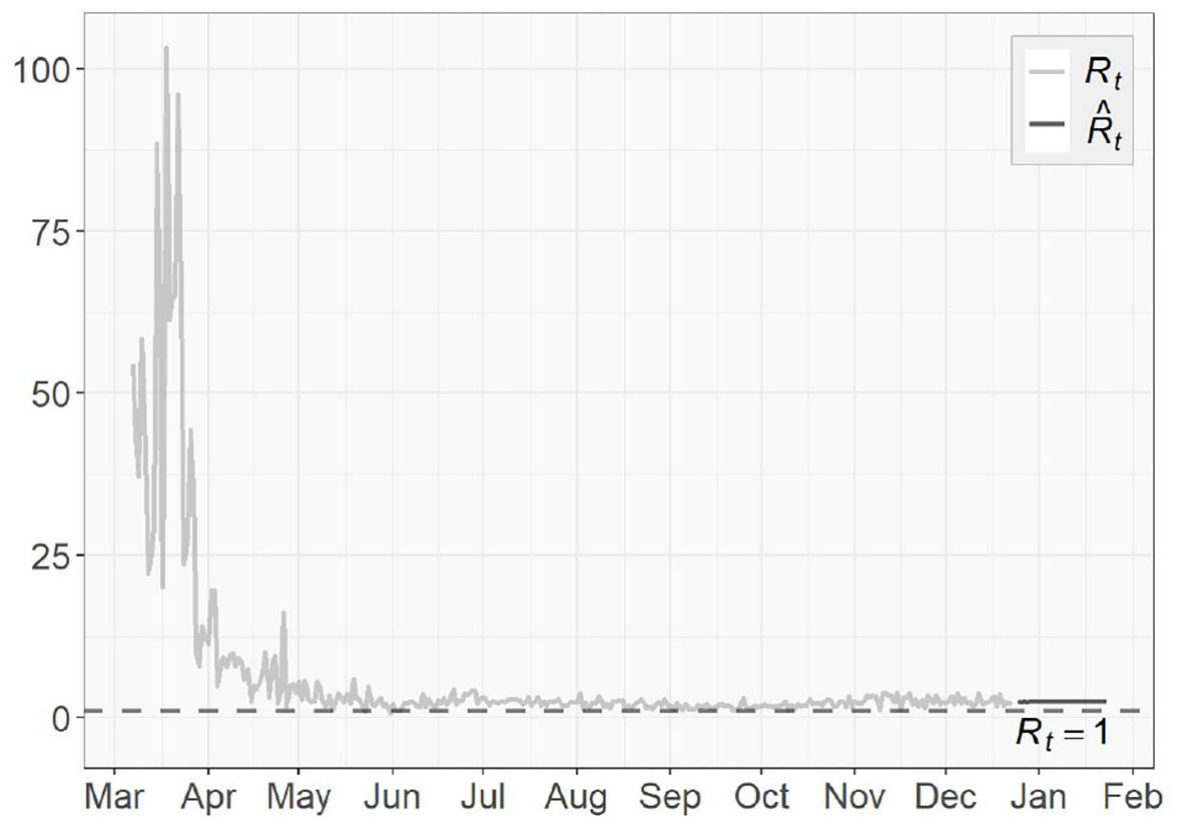
Time-dependent basic reproduction number for the United States.

**Figure 12:**
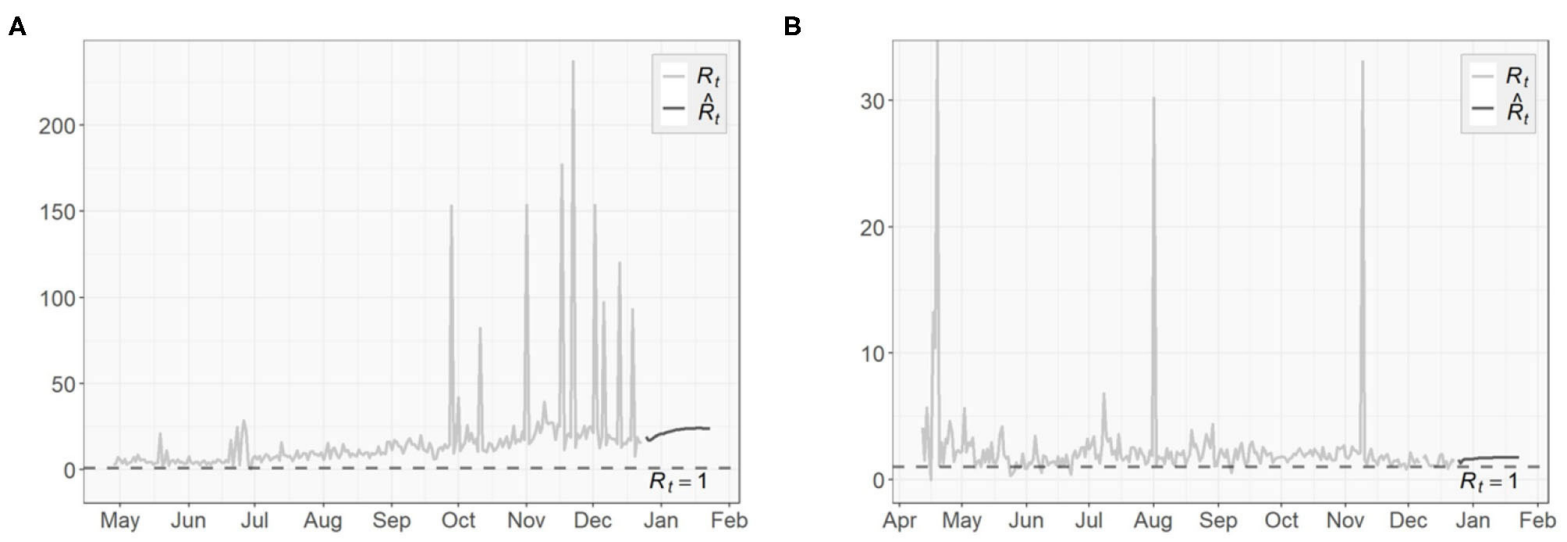
Time-dependent basic reproduction number for **(A)** New York **(B)** North Dakota.

To assess the spread of COVID-19, we also obtain the 1-day prediction for the time-dependent basic reproduction number $R_{t}$ using (31). The results for the three regions are presented in Figures 11, 12, with horizontal lines representing $R_{t}=1$. As discussed in section 3.3, the virus will decline and gradually die out when $R_{t}<1$. Otherwise, it will continue to spread. According to the results shown in Figures 11, 12, only very few points fall below the horizontal line, while the majority lies above it. For NY, the surge in fall, 2020 and some scattered large values agree with the increasing trends in both the confirmed cases and the transmission rate we see in Figures 4, 6, respectively.

The basic reproduction numbers $R_{t}$ for each of the next 30 days are estimated to be >1 for all three regions. The means of predicted values are found to be 2.48 for US, 22.28 for NY and 1.68 for ND, which suggests the inflection point, where $R_{t}$ stabilizes below 1 afterwards, has not been reached yet, especially for the NY case, where instead of having a decreasing trend, an increasing $R_{t}$ actually emerges over time. For US and ND, the curves gradually approaching the horizontal lines of $R_{t}<1$ indicates that the measures taken to tackle the pandemic are taking effect, but at this point it is sill too early to relax them.

### 4.4. Simulation Results for the SEIR and SEVIS Models

We also simulate the long-term development of the COVID-19 pandemic based on SEIR and SEVIS models. March 17, 2020, the first day in our US data, is chosen as the starting date of the pandemic in the simulations.

For the SEIR model, we set the transition rate to $\sigma_{t}=\frac{1}{5.1}$ according to Table 1. To simulate as close to the reality as possible, we set the transmission rate β_*t*_ and the recovery rate γ_*t*_ to the means of their true value series obtained in section 4.1. To construct the initial conditions of the system, we use the initial values *I*(0) = 311 and *R*(0) = 27 obtained from the data as well. In previous studies, the average Infected-Suspected ratio in China, one of the earliest hot spots of the global COVID-19 outbreak, was found to be 2.399 (e.g., Fairoza Amira et al., 2020). In this simulation, due to the lack of data of the exposed group, we use the same ratio to initialize *E*(*t*), i.e., $E\left(0\right)=\frac{1}{2.399}I\left(0\right)\approx 130.$ According to the U.S. and World Population Clock (United States Census Bureau, 2020), the U.S. population is *N* = 329, 227, 746. Using (5), we have: *S*(0) = *N* − *E*(0) − *I*(0) − *R*(0) ≈ 329, 227, 278.

**Figure 13:**
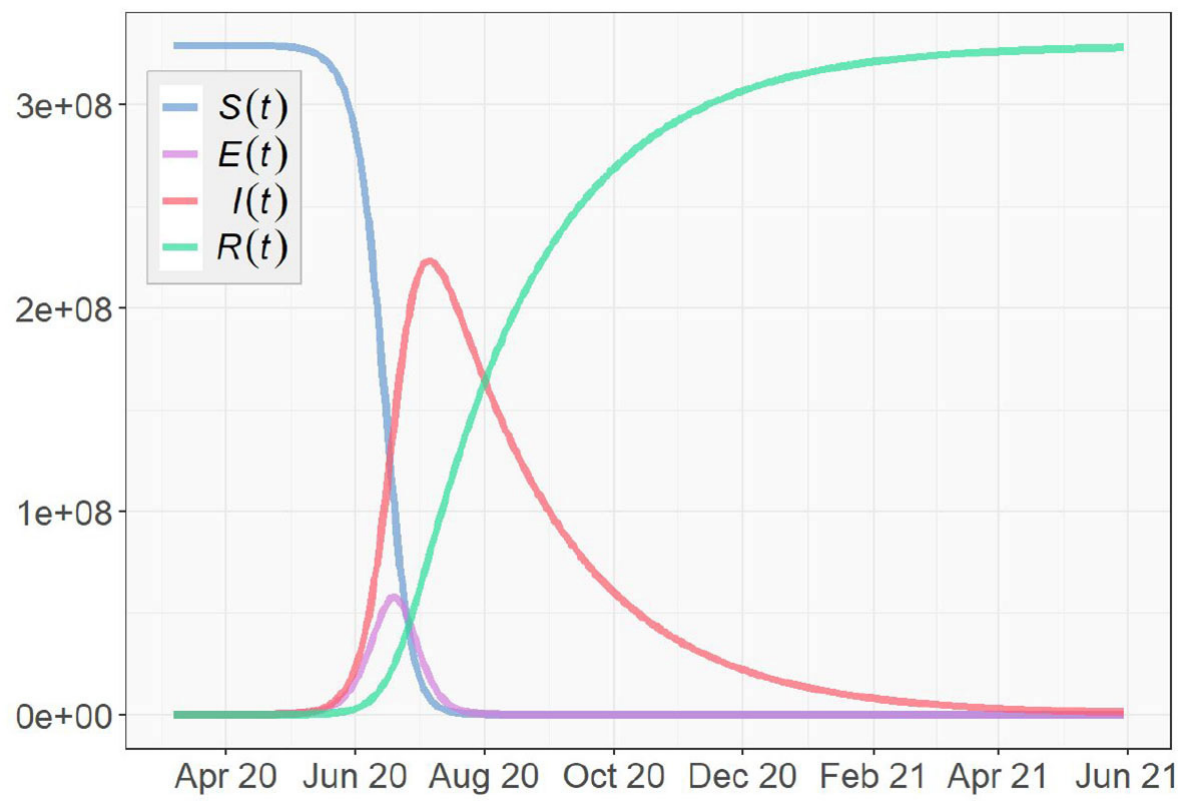
Simulation based on the SEIR Model for the U.S.

With the aforementioned parameter settings and initial conditions, we simulate the COVID-19 pandemic for the US. As shown in Figure 13, the number of infected people reaches a peak in early July, 2020, and the pandemic gradually dies out in summer 2021. It is important to note that the simulation is only theoretical and restricted by given conditions. These conditions can be dramatically different in realty. Moreover, no mitigation measure of any kind that can possibly prevent or limit the spread of the virus is considered in the simulation, such as wearing facial coverings, social distancing, community lockdowns, and work-from-home policies. Being free of the influences of such factors indicates that the pandemic might develop slower in the simulation than in reality. Since many states of the U.S. are following the strict guidelines set by CDC, the pandemic is highly likely to end earlier than the simulation result.

Next, we take immunity, reinfection and vaccination into account, and simulate the pandemic according to the SEVIS model proposed in section 3.1. The parameter settings of β_*t*_, σ_*t*_, and γ_*t*_ remain the same as in the SEIR simulation. For the vaccination rate *v*_*t*_, we clarify a starting date of vaccination *t*_*v*_. Before the vaccination starts, i.e., for *t* < *t*_*v*_, *v*_*t*_ = 0. When *t* ≥ *t*_*v*_, *v*_*t*_ becomes positive and based on the discussion in section 3, we assume *v*_*t*_ to start at a low value in realty and exponentially increase as time goes on. Here, we simplify this process by assuming the mean of {*v*_*t*_|*t* ≥ *t*_*v*_} to be 1% per day and assigning it to *v*_*t*_, and let the vaccination start on January 1, 2021. As for the last parameter *w* in Table 1, the fraction of infected cases that become immune after recovery is currently unknown. In this simulation, we assume *w* to be 0.5.

**Figure 14:**
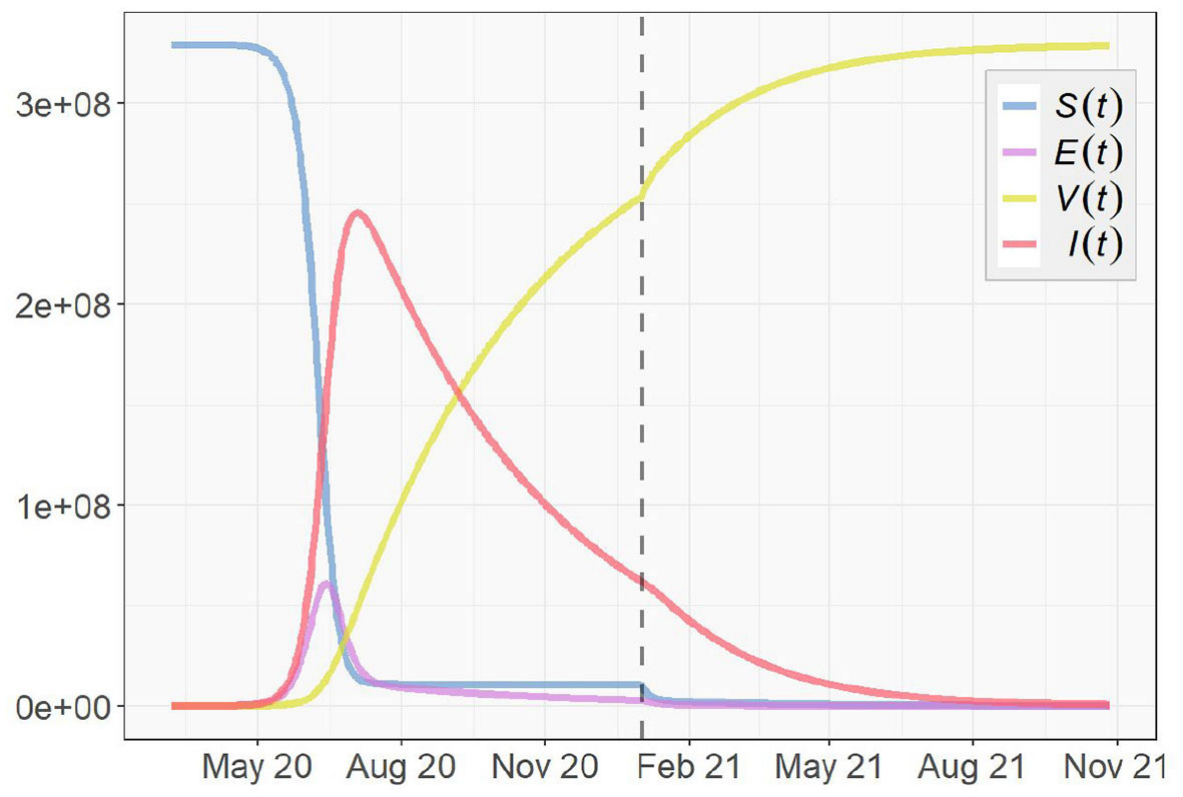
Simulation based on the SEVIS Model for the U.S.

Figure 14 shows the simulation result with the vertical dashed line representing *t* = *t*_*v*_, (i.e., the first day of 2021). We notice that the trajectories obtained from the SEVIS model before the vaccination are nearly identical to the previous SEIR simulation. Once vaccination begins, the growth of the immunity group *V*(*t*) and the decrease of the infected group *I*(*t*) clearly accelerate. However, different from SEIR model which assumes no reinfection, the SEVIS model does allow reinfection, which leads to a longer time for the virus to die out. To speed up the process, we can employ a larger value for *w*, i.e., increased flows from *I*(*t*) to *V*(*t*) and reduced flows from *I*(*t*) to *S*(*t*).

## 5. Conclusion

Considering the incubation period of COVID-19, we first proposed a time-dependent SEIR model with the time-dependent parameters estimated by LASSO regression. The proposed model is validated using the national level data (the United States) and state level data (New York and North Dakota). Overall, our proposed model outperforms the SIR model with smaller prediction errors. Furthermore, by taking immunity, reinfection, and vaccination into account, we proposed a time-dependent SEVIS model without assuming guaranteed immunity after recovery as in the SEIR model. Simulations are performed using the proposed two models to predict the spread of COVID-19 pandemic for the United States.

With the daily recorded data in the U.S., our algorithm predicts that the numbers of the infected and recovered individuals will keep increasing at a high rate in the short future. The total number of confirmed cases in the U.S. is estimated to reach close to 25.7*M* by late January, 2021, while North Dakota and New York will face 1.26 and 0.96*M* total confirmed cases, respectively. Given the historical transmission and recovery rate of the COVID-19, the simulation of SEVIS model predicts that the pandemic will die down in fall 2021, assuming the mean vaccination rate to be 1% per day and the probability of gaining immunity after recovery to be 50%. Note that this prediction is subject to change with more accurate parameters chosen according to the real data once vaccination starts.

In addition, it is crucial to understand that neither of the prediction and simulation takes any mitigation measures that can prevent or limit the growth of the pandemic into consideration, such as social distancing, facial covering, lockdown restrictions, and closing non-essential businesses. As a result, the end of the pandemic in reality is highly likely to come earlier than the numeric outcome. However, at this point the spread of the pandemic is still ongoing and has not been contained yet, as the time-dependent basic reproduction number for US is still steadily positive. Also, in some particular parts of US (e.g., New York), a new surge in the transmission rate was detected as the end of the year 2020 approaches. These all could serve as an alert that it is too early to relax the measures already implemented to tackle the pandemic. Fortunately, these measures have been proven effective by evidences. We expect them to continue taking effect over time and suggest the necessity of bring in more. Hopefully, with effort made by people around the world and the upcoming release of vaccine, we will be able to conquer this global crisis in no time.

Another limitation of the proposed time-dependent SEVIS model is that, it assume absolute immunity to the virus after vaccination, while in reality, the effectiveness of the vaccine is not 100% guaranteed. For example, as reported by the BBC news, a single dose of the Moderna vaccine can provide 80.2% protection. When a second dose is injected after a period of time, the effectiveness rise to 95.6%. In the future, we would like to extend the model by factoring in changing effectiveness at different stage of the vaccination.

## Data Availability Statement

The original contributions presented in the study are included in the article/supplementary material, further inquiries can be directed to the corresponding author/s.

## Author Contributions

JZ designed the study. YL collected data for analysis and interpreted the results and drafted the manuscript. JZ and YL analyzed the data and developed the models. LG, YZ, XC, and JZ revised the manuscript. All authors gave final approval for publication.

## Conflict of Interest

The authors declare that the research was conducted in the absence of any commercial or financial relationships that could be construed as a potential conflict of interest.

## Footnotes

^1^T cells are one of the important white blood cells of the immune system, and play a central role in the adaptive immune response.

## References

[B1] Chen Y.-C. Lu P.-E. Chang C.-S. Liu T.-H. (2020). A time-dependent sir model for covid-19 with undetectable infected persons. IEEE Trans. Netw. Sci. Eng. 7, 3279–3294. 10.1109/TNSE.2020.3024723 PMC8769021 37981959

[B2] Diekmann O. Heesterbeek J. A. P. Metz J. A. J. (1990). On the definition and the computation of the basic reproduction ratio r0 in models for infectious diseases in heterogeneous populations. J. Math. Biol. 28, 365–382. 10.1007/BF00178324 2117040

[B3] Dong E. Du H. Gardner L. (2020). An interactive web-based dashboard to track covid-19 in real time. Lancet Infect. Dis. 20, 533–534. 10.1016/S1473-3099(20)30120-1 32087114 PMC7159018

[B4] Fairoza Amira B. H. Cher H. Hafeez N. Dominic L. Guanhua L. Mohammad S.. (2020). Coronatracker: World-Wide Covid-19 Outbreak Data Analysis and Prediction. Bulletin of the World Health Organization.

[B5] Ganyani T. Kremer C. Chen D. Torneri A. Faes C. Wallinga J.. (2020). Estimating the generation interval for covid-19 based on symptom onset data, March 2020. Eurosurveilance 25:2000257. 10.2807/1560-7917.ES.2020.25.17.2000257 32372755 PMC7201952

[B6] Gao D. Porco T. C. Ruan S. (2016). Coinfection dynamics of two diseases in a single host population. J. Math. Anal. Appl. 442, 171–188. 10.1016/j.jmaa.2016.04.039 27667856 PMC5032845

[B7] Hsieh F. Zheng J. (2019). Unraveling pattern-based mechanics defining self-organized recurrent behaviors in a complex system: a zebrafish's calcium brain-wide imaging example. Front. Appl. Math. Stat. 5:13. 10.3389/fams.2019.00013

[B8] Katul G. G. Mrad A. Bonetti S. Manoli G. Parolari A. J. (2020). Global convergence of COVID-19 basic reproduction number and estimation from early-time sir dynamics. PLoS ONE 15:e239800. 10.1371/journal.pone.0239800 32970786 PMC7514051

[B9] Murray C. J. (2020). Forecasting COVID-19 impact on hospital bed-days, icu-days, ventilator-days and deaths by us state in the next 4 months. medRxiv [Preprint]. 10.1101/2020.03.27.20043752

[B10] Ngonghala C. N. Iboi E. Eikenberry S. Scotch M. MacIntyre C. R. Bonds M. H.. (2020). Mathematical assessment of the impact of non-pharmaceutical interventions on curtailing the 2019 novel coronavirus. Bellman Prize Math. Biosci. 325:108364. 10.1016/j.mbs.2020.108364 32360770 PMC7252217

[B11] Read J. M. Bridgen J. R. Cummings D. A. Ho A. Jewell C. P. (2020). Novel coronavirus 2019-nCoV: early estimation of epidemiological parameters and epidemic predictions. medRxiv [Preprint]. 10.1101/2020.01.23.20018549 PMC8165596 34053269

[B12] Schmidt W. (1981). Eisen, M.: Mathematical models in cell biology and cancer chemotherapy. Lecture notes in biomathematics, vol. 30. Springer-Verlag, Berlin-Heidelberg-New York 1979. IX, 431 s., 70 abb., 17 tab., DM 39,-. Biometr. J. 23, 519–520. 10.1002/bimj.4710230517

[B13] Sharomi O. Gumel A. (2011). Dynamical analysis of a sex-structured chlamydia trachomatis transmission model with time delay. Nonlin. Anal. Real World Appl. 12, 837–866. 10.1016/j.nonrwa.2010.08.010

[B14] Shen M. Peng Z. Xiao Y. Zhang L. (2020). Modelling the epidemic trend of the 2019 novel coronavirus outbreak in china. Innovation 1:100048. 10.1016/j.xinn.2020.100048 PMC7831648 33521762

[B15] Tan W. Lu Y. Zhang J. Wang J. Dan Y. Tan Z.. (2020). Viral kinetics and antibody responses in patients with covid-19. medRxiv [Preprint]. 10.1101/2020.03.24.2004238232950003

[B16] To K. K. W. Tsang O. T. Y. Leung W. S. Tam A. R. Wu T. C. Lung D. C.. (2020). Temporal profiles of viral load in posterior oropharyngeal saliva samples and serum antibody responses during infection by sars-CoV-2: an observational cohort study. Lancet Infect. Dis. 20, 565–574. 10.1016/S1473-3099(20)30196-1 32213337 PMC7158907

[B17] Toda A. A. (2020). Susceptible-infected-recovered (sir) dynamics of COVID-19 and economic impact. arXiv [Preprint] arXiv:2003.11221.

[B18] United States Census Bureau (2020). U.S. and World Population Clock. United States Census Bureau. Available online at: https://www.census.gov/ (accessed December 24, 2020).

[B19] van den Driessche P. Watmough J. (2002). Reproduction numbers and sub-threshold endemic equilibria for compartmental models of disease transmission. Bellman Prize Math. Biosci. 180, 29–48. 10.1016/S0025-5564(02)00108-6 12387915

[B20] Wu T. Ge X. Yu G. Hu E. (2020). Open-source analytics tools for studying the COVID-19 coronavirus outbreak. medRxiv [Preprint]. 10.1101/2020.02.25.20027433

[B21] You C. Deng Y. Hu W. Sun J. Lin Q. Zhou F.. (2020). Estimation of the time-varying reproduction number of COVID-19 outbreak in china. Int. J. Hyg. Environ. Health 228:113555. 10.1016/j.ijheh.2020.113555 32460229 PMC7211652

[B22] Zheng J. Fushing H. Ge L. (2019). A data-driven approach to predict and classify epileptic seizures from brain-wide calcium imaging video data. IEEE/ACM Trans. Comput. Biol. Bioinformatics 17, 1858–1870. 10.1109/TCBB.2019.2895077 30676975

[B23] Zheng J. Liang M. Ekstrom A. D. Ge L. Yu W. Hsieh F. (2018). On association study of scalp EEG data channels under different circumstances, in International Conference on Wireless Algorithms, Systems, and Applications (New York, NY), 683–695. 10.1007/978-3-319-94268-1_56

